# Improving the CRCC-DHR Reliability: An Entropy-Based Mimic-Defense-Resource Scheduling Algorithm

**DOI:** 10.3390/e27020208

**Published:** 2025-02-16

**Authors:** Xinghua Wu, Mingzhe Wang, Yun Cai, Xiaolin Chang, Yong Liu

**Affiliations:** 1 School of Cyberspace Science and Technology, Beijing Jiaotong University, Beijing 100044, China; 22115141@bjtu.edu.cn; 2 Institute of Computing Technology, China Academy of Railway Sciences, Beijing 100081, China; liuyong1@rails.cn; 3 School of Information Science and Technology, Southwest Jiaotong University, Chengdu 610031, China; zhouxiaoxia@swjtu.edu.cn

**Keywords:** China Railway Cloud Center, DHR architecture, entropy, game theory, mimic defense, scheduling

## Abstract

With more China railway business information systems migrating to the China Railway Cloud Center (CRCC), the attack surface is expanding and there are increasing security threats for the CRCC to deal with. Cyber Mimic Defense (CMD) technology, as an active defense strategy, can counter these threats by constructing a Dynamic Heterogeneous Redundancy (DHR) architecture. However, there are at least two challenges posed to the DHR deployment, namely, the limited number of available schedulable heterogeneous resources and memorization-based attacks. This paper aims to address these two challenges to improve the CRCC-DHR reliability and then facilitate the DHR deployment. By reliability, we mean that the CRCC-DHR with the limited number of available heterogeneous resources can effectively resist memorization-based attacks. We first propose three metrics for assessing the reliability of the CRCC-DHR architecture. Then, we propose an incomplete-information-based game model to capture the relationships between attackers and defenders. Finally, based on the proposed metrics and the captured relationship, we propose a redundant-heterogeneous-resources scheduling algorithm, called the Entropy Weight Scheduling Algorithm (REWS). We evaluate the capability of REWS with the three existing algorithms through simulations. The results show that REWS can achieve a better reliability than the other algorithms. In addition, REWS demonstrates a lower time complexity compared with the existing algorithms.

## 1. Introduction

**Figure 1:**
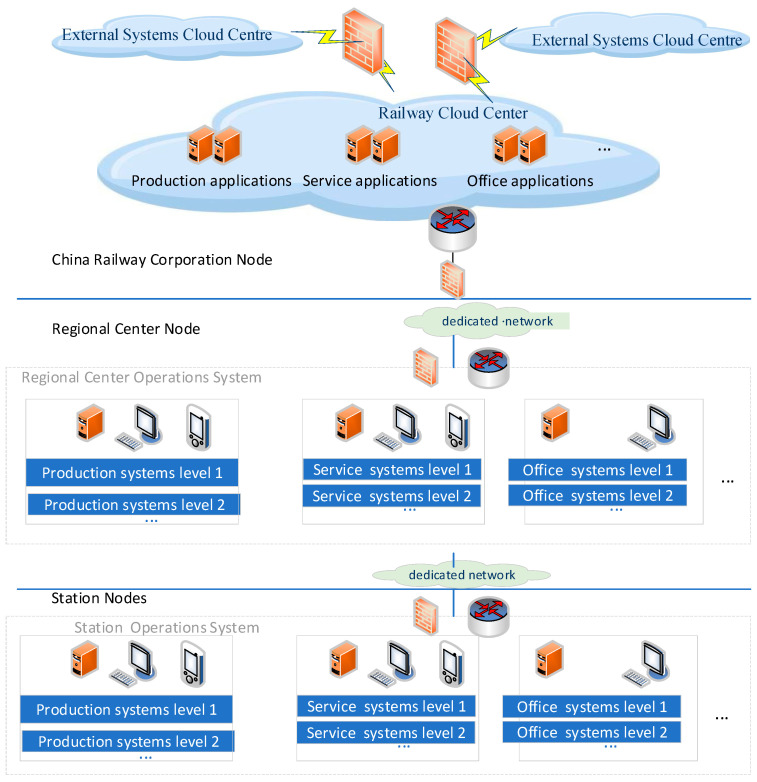
The architecture of the CRCC.

The China Railway Cloud Center (CRCC) is primarily responsible for the construction and deployment of various business information systems supporting China Railway internal services, production, management, and office functions to various end nodes [1]. These nodes may be the China Railway Corporation nodes, regional center nodes, or station-segment nodes [1]. Figure 1 illustrates the CRCC architecture, characterized by the deployment of unified computing resources and unified security protection resources at the central nodes of the corporation. The CRCC should meet the security requirements of the regional center nodes and station-segment nodes when these nodes access various business systems deployed in the CRCC. This is achieved by implementing unified security protection and access aggregation measures at the network edges of these nodes. These protection measures ensure secure data access across different application domains.

The past years have witnessed a surge in the number and sophistication of zero-day vulnerabilities, which pose a critical threat to organizations of all sizes [2], and an increase in APT attacks on the critical infrastructures, such as public transportation and electricity [3,4,5]. As the scale of information system construction in the CRCC has continuously expanded, the risk of various attacks is becoming even more severe. There are the following two main reasons.

(1) The attack surface of various application systems is growing [6]. As various production, offices, and service systems of the railway rapidly expand, the types of intelligent terminals at regional center nodes and station-segment nodes keep being diversified. Additionally, the stations accommodate many more individuals, through which attacks may be carried out on the CRCC if these individuals are not protected well. This leads to a gradual increase in the overall attack surface of the CRCC systems. As a result, the probability of attack occurrence is continuously rising.
(2) Artificial Intelligence (AI) technologies and advancements are being used in cyber attacks [7,8]. The attacks can apply the knowledge memorization of previous attacks to make adaptive adjustment to the defense strategy, thus making the attack more persistent, covert, and not easy to counter.

Techniques of addressing security and reliability issues include Moving Target Defense (MTD) [9,10,11], Cyber Mimic Defense (CMD) [12,13], Byzantine Fault Tolerance [14,15,16], and Redundancy Fault Tolerance [17,18] technologies. The characteristics of these technologies are summarized in Table 1.

Among the techniques mentioned, Cyber Mimic Defense (CMD) technology is one of the prevailing methods. It aims to make the targeted system uncertain and dynamic in time and space so as to effectively counter potential attacks. CMD technology constructs a Dynamic Heterogeneous Redundancy (DHR) architecture. This architecture introduces the fault-tolerant features of dynamism, heterogeneity, and redundancy into the system. It also introduces closed-loop feedback features into the system. These introduced features enhance the system’s robustness and intrinsic security [19,20,21]. Compared with other technologies, CMD offers more flexibility in the architectural pattern and fault-tolerant form. As a result, CMD technology significantly enhances the security and reliability of systems in various complex scenarios.

Studies and the application of CMD technology have demonstrated its effectiveness in addressing endogenous security problems. However, there are at least two weaknesses in applying this technology within the CRCC.

**Weakness 1**: The existing research assumes that there are infinite schedulable heterogeneous resources in the cloud environment.

**Weakness 2**: They assume that there is no information entropy decay in the redundancy scheduling process. This decay makes the entire CMD system unable to guarantee its reliability when there exist memorization-based attacks. By memorization, we mean that the adversary can apply the information, which the adversary obtains in the previous attacks, to the later attacks. But this decay exists when there is a finite number of schedulable resources and there exists memorization-based attacks.

This paper aims to deal with these two weaknesses. The main contributions are listed as follows.

(**Contribution 1**) We propose three reliability assessment metrics for the CRCC-DHR architecture under the conditions of limited schedulable heterogeneous resources and memorization-based attacks. The metrics are detailed in Section 3, including the number of scheduling states of the redundancy resources (executors), the information entropy value, and the decay rate of the information entropy value. These metrics are based on the information, which can be obtained by the adversary after a successful attack and will be applied to the subsequent attacks.

(**Contribution 2**) We propose an incomplete-information-based game model to capture the relationships between attackers and defenders for the DHR architecture under the conditions of limited schedulable resources and memorization-based attacks. We also apply information entropy to solve the model and then derive the condition that should be satisfied in order to guarantee the maximum attacker–defender equilibrium gain.

(**Contribution 3**) We propose an information-entropy-weight-based redundant executor scheduling algorithm. We firstly define the reliability maximization model, where we define the objective function based on the metrics in **Contribution 1**, and we construct the constraints based on both the metrics in **Contribution 1** and the game model developed in **Contribution 2**.

We perform simulations to evaluate the proposed algorithm’s capability by comparing it with three existing algorithms, with respect to the traditional scheduling cycle metrics as well as the metrics proposed in this paper.

The subsequent parts of this paper are organized as follows. Related work is presented in Section 2. The reliability index model under the conditions of limited schedulable resources and with a memorization-based attack is introduced in Section 3. The game theory modelling method and the scheduling algorithm are introduced in Section 4. Experimental simulations are carried out in Section 5, and the conclusions of this paper and outlooks of the future work are presented in Section 6. 

The variables in the article and the symbols of the formulas are shown in Table 2.

## 2. Related Work

**Figure 2:**
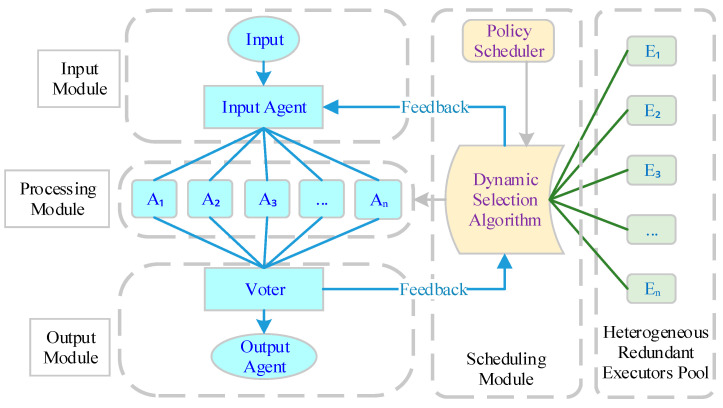
Dynamic Heterogeneous Redundancy model structures.

The DHR architecture [12,13] is the core architecture of the CMD, including the components of input agents, executors, voters, policy schedulers, and a pool of heterogeneous redundant executors, as illustrated in Figure 2. The fundamental processing flow of the system is as follows:

(i) Dynamically Allocating Redundant Executors: The scheduling module dynamically assigns redundant executors from the pool to the processing module using a dynamic selection algorithm.
(ii) Forwarding User-Sent Message Status: The input agent forwards the status of the messages sent by the user to different redundant executors within the processing module.
(iii) Processing and Making a Consistent Decision: The redundant executors process the received requests and send them to the voting unit. The voting unit then makes a consistent decision and produces the output result.
(iv) Negative Feedback and Redundant Executors Rescheduling: If any inconsistent rulings are detected during the decision-making process, the redundant resources/executors that are responsible receive negative feedback. This feedback is sent back to the scheduling module, which triggers the rescheduling of the redundant executors.

There exists research on mimic defense architectures for public and private cloud networks. Li et al. [22] proposed a mimic cloud security architecture called Mimi-cloud for 5G core networks. This architecture enhances the security of 5G core networks by uniformly computing the heterogeneous vector metrics of container cloud environments. It also improves security through unified Kubernetes scheduling and the cross-checking of container cloud executables. Wu et al. [23] introduced an active defense development framework for cloud-native environments. They used technologies such as multi-version assembly, multi-instance deployment, and diversified compilation to increase the system complexity and then improve its ability to resist attacks. Wang et al. [24] proposed an IoT DHR architecture based on the double deep reinforcement learning network (DDQN). They used the DDQN network to train and optimize scheduling and decision strategies. This enables dynamic scheduling in container cloud environments driven by Kubernetes. Sepczuk [25] developed a defense model that combines the DHR architecture with a WAF firewall in a cloud environment. The WAF firewall establishes temporary redundant execution rules when potential HTTP attacks are detected, thereby enhancing the security of the system.

Regarding the optimization of mimic defense strategies, the relevant research primarily uses various modelling techniques, which mathematically model and simulate the dynamic scheduling strategies of redundant executors. These modelling techniques include probability models, game models, and information entropy models, which are detailed in the following.

(i) Studies based on probabilistic modeling.

This type of study is a classical approach. Through the probabilistic model, researchers optimize the probability of a successful attack on a redundant executor. They use the heterogeneity of the executor as a metric to assess the likelihood of an attack. Based on this assessment, they develop relevant scheduling strategies.

Chen et al. [26] addressed the nonlinear problem of component heterogeneity superposition. They introduced a heterogeneous evaluation model based on the minimum L-order error probability. Li et al. [27] proposed several scheduling algorithms, including a time threshold-based TIRTS scheduling algorithm, a task-based threshold TARTS scheduling algorithm, and the MQS multi-level queue scheduling algorithm. Zhu et al. [28] developed a comprehensive scheduling algorithm called HHAC. This algorithm is based on high-order heterogeneity and adaptive historical confidence. It aims to optimize the dynamic strategy of the DHR architecture. They also analyzed the dynamic indicators of the CRS, TIRTS, RSMS, and HHAC algorithms. Shao et al. [29] proposed a dynamic scheduling algorithm called HCDC. This algorithm is based on historical credibility and K-Means heterogeneous clustering. Through simulation experiments, they compared the HCDC algorithm with the RS, MD, and OMD algorithms in terms of attack rates and other indicators.

(ii) Studies based on game model and information entropy model.

Research based on the game model focus on analyzing the gain indicators for both attacking and defending parties within the DHR architecture. This involves examining the system’s response to operational events. The goal is to improve the system reliability by optimizing the game strategies of both the attackers and defenders. Research using the information entropy model, on the other hand, assesses system reliability by monitoring changes in information entropy during the operation of the mimic defense system.

Hu et al. [30] analyzed the heterogeneity of redundant executors and the probability of being attacked based on the information entropy theory. They proposed a defense chain model incorporating information entropy and heterogeneity. The numerical analysis of the attack success rate was conducted using the successive Markov model to verify the effectiveness of the DHR architecture. Chen et al. [31] proposed a dynamic architecture evaluation method based on incomplete information game strategies. They used the Markov chain model to calculate and evaluate the benefits for both offense and defense. This approach was used to verify the security of the architecture. Shi et al. [32] developed an evolutionary DHR system. They addressed the issue of a limited number of heterogeneous executors by introducing evolutionary sub-strategies for the executors. The effectiveness of their proposed scheme was verified through the construction of a game model. Hu et al. [33] used the static game theory to explore the unique Nash equilibrium within the DHR architecture. They applied the Adam algorithm to analyze and validate the dynamics, heterogeneity, and failure rates affecting the DHR architecture in detail. Shao et al. [34] proposed an active defense method, which exploited adaptive anomaly sensing for the mimic IoT. This method aimed to deal with uncertain threats, such as known vulnerabilities and backdoors existing within the IoT, which were difficult for traditional passive network security technologies to effectively counter.

In summary, there has been extensive research on the application of mimic defense architectures in cloud environments and the optimization of defense strategies. However, issues remain in two key areas of related research:

(i) Research on the mimic defense architecture in cloud-centric environments often assumes that there are sufficient schedulable heterogeneous resources. Currently, relevant studies mainly focus on leveraging Kubernetes as the management and scheduling center within the cloud center system architecture. Kubernetes is used for the unified and rapid deployment of container cloud environments and integrates with the DHR architecture to enhance system reliability. However, there is limited research on the reliability of private cloud centers as unified carriers for multi-system multiplexing. These centers typically have more uniform resources to be allocated and insufficient scheduling heterogeneity.
(ii) There is less research on the reliability of mimetic scheduling strategies against memorization-based attacks. Currently, research on mimic defense scheduling strategies mainly addresses none-memorization-based attacks. These attackers do not focus on specific targets or analyze the attack environment after the attack. In such cases, the system’s long-term reliability can be maintained through scheduling and cleaning strategies for the redundant executors. However, the situation is different for those cases where there exist attacks with clear targets and 0-day vulnerability attacks. Traditional scheduling methods cannot fully clarify these threats in the short term. That is, there is a lack of research on the reliability of mimetic scheduling strategies under these conditions.

In our previous research [35], we modeled and analyzed the reliability-related metrics of the DHR architecture for application-oriented systems based on failure probability modeling in the CRCC. The process is as follows:

(i) We consider a mimic defense system *A* with a total redundancy of n and m heterogeneous redundant executors that can be dispatched at a time, denoted as ${\left\{a_{1}\ldots a_{m}\right\}}_{n}$, and make the following assumptions:

**Assumption** **1.** *The reliability factors affecting system A include only system failures due to attacks on the redundant executors and do not include other failure factors.*

**Assumption** **2.** *When an adversary launches an attack, there is at most only a single redundant executor to fail, and the attack is an independent event.*

The system reliability *R*(*A*)*_m_* can be expressed by its failure probability function *p*. That is, as shown in Equation (1), where *p*(*A*)*_m_* denotes the failure probability of the proposed defense system *A* and $p{\left(a_{i}\ldots a_{j}\right)}_{j-i}$ denotes the joint distribution probability of *l-i* redundant executors failing together.

(1) $$R{\left(A\right)}_{m}=1-p{\left(A\right)}_{m}=1-\sum_{l-i=[(m+1)/2]}^{m}p{\left(a_{i}\ldots a_{l}\right)}_{l-i}$$

(ii) We further set *n* redundant executors, each of which consists of some different components. For any two redundant executors, there is often a certain degree of homomorphic similarity between the different components. The higher the degree of similarity of the components or the higher the number of similar components, the higher the probability that a common-mode vulnerability will lead to the failure of the two redundant executors. The system failure probability can be further expressed in Equation (2):

(2) $$p{\left(A\right)}_{m}=\sum_{l-i=[(m+1)/2]}^{m}p{\left(a_{i}\ldots a_{l}\right)}_{l-i}=\sum_{l-i=[(m+1)/2]}^{m}p(a_{i})p{\left(a_{i+1}\ldots a_{l}|a_{i}\right)}_{l-i-1}$$

Also, noting that $s_{ij}$ denotes the similarity of any two redundant executors $a_{i},a_{j}$, the system *A* similarity can be represented by the matrix *S* as follows:

(3) $$S=\left(\begin{array}{ccc} 1 & \ldots & s_{1n}\\ \vdots & \ddots & \vdots \\ s_{n1} & \cdots & 1 \end{array}\right)$$

If the relationship between the similarity and the probability of an attack on a redundant executor is expressed as $f(s_{ij})$, then the probability $p(a_{j}|a_{i})$ of the redundant executor *a_i_* being successfully attacked can be expressed by Equation (4).

(4) $$p(a_{j}|a_{i})=p(a_{j})\cdot f(s_{ij})$$

Then, for system *A*, the failure probability *p*(*A*) under the majority consensus decision condition, i.e., the number of failed redundancies *k* satisfies *k* ≥ [(*m* + 1)/2], can be expressed by Equation (5).

(5) $$p{\left(A\right)}_{m}=\sum_{k=[\frac{m+1}{2}]}^{m}\prod_{i=1,j\neq i}^{k}p(a_{i})p(a_{j})f(s_{ij})$$

Through the above analysis, it can be concluded that there are two key factors affecting the failure probability of the DHR architecture, i.e., the probability $p(a_{i})$ of redundant executors being attacked, and the similarity mapping function $f(s_{ij})$ between the redundant executors. $p(a_{i})$ is related to the security of each component of the system, which is a relatively fixed attribute. $f(s_{ij})$ is related to the size of the entire resource pool of the cloud center, and the type and number of heterogeneous components. Since cloud environments are often built uniformly in practice, the number of heterogeneous resources that can be scheduled is very limited, and thus the range in the variation of the two attributes $p(a_{i})$ and $f(s_{ij})$ is also limited, which does not allow for an effective assessment of the reliability of the CRCC-DHR architecture under the condition of limited schedulable resources.

This paper considers the CRCC-DHR where there are limited schedulable heterogeneous resources and various targeted memorization-based attacks from external networks. We use the information entropy model to perform the modeling and analysis. Additionally, we propose a redundancy scheduling algorithm based on random entropy weights and the game model. This algorithm aims to improve the reliability of the mimic defense architecture in environments with limited scheduling resources and memorization-based attacks.

## 3. Three Metrics for Assessing the Reliability of the CRCC-DHR Architecture

The discussions in Section 2 indicate that it is hard to assess the reliability of CRCC-DHR architectures well under the conditions of limited heterogeneous resources and memorization-based attacks using the failure probability approach [35]. Therefore, this section proposes information-entropy-based metrics to analyze the reliability of the CRCC-DHR architecture.

**Figure 3:**
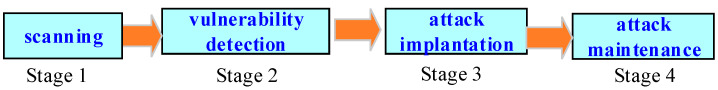
Attack chain.

We consider the common attack chain for a system attack, including four stages: scanning, vulnerability detection, attack implantation, and attack maintenance, as shown in Figure 3.

For memorization-based attacks, the adversary often first scans and sniffs the target host and then implants an attack agent on the system through 0-day vulnerabilities. When the attack is blocked at any stage, the attacker will record the blocked state and then resume scanning and sniffing in the subsequent attacks.

It is known that the reliability of the redundant system *A* stems from the uncertainty of being attacked [30]. The greater the uncertainty, the more effort an attacker must employ, resulting in a higher system reliability. This uncertainty can be measured by the system’s information entropy *H*, which is the expected value of the uncertainty probability for each redundant executor within the system. That is, for a system $X=\{x_{t}|x_{1}\ldots x_{l}\}$ containing *l* executors, its total information entropy can be expressed by Equation (6).

(6) $$H(X)=E(F(p_{xt}))=-\sum_{t=1}^{l}p_{xt}\log p_{xt}$$

where $p_{xt}$ denotes the probability that $x_{t}$ pieces of information are available, and $F(p_{xt})$ denotes an uncertain function of the probability of the occurrence of $x_{t}$.

**Figure 4:**
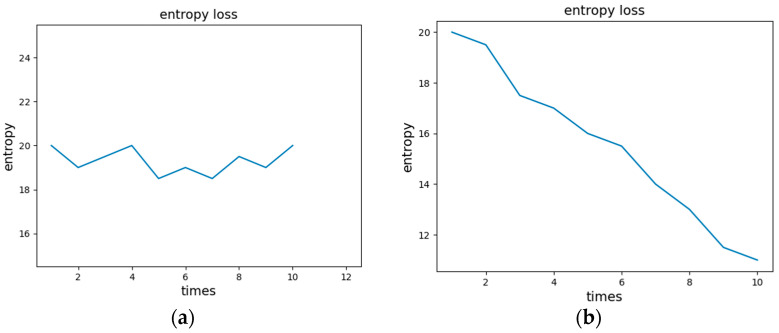
(**a**) Trend of information entropy loss during the scheduling of redundant systems under infinite resources. (**b**) Trend of information entropy loss during the scheduling of redundant systems under finite resources.

We use *H*(*A*) to denote the initial total entropy of the mimetic defense system *A*. Suppose there exists a 0-day vulnerability in *A*, which cannot be eliminated through offline cleaning for the time being. Then, under the condition of infinite redundant executor resources, the defender can force the attacker to repeat between states 1 and 2 by continuously performing new redundant executors’ scheduling. That is, in the information entropy model, a single redundant executor can be attacked to make the entropy decrease. But when *n* tends towards infinity, the total information entropy of the system still remains undiminished. In addition, under the condition of finite redundant resources/executors, although the defender can conduct repeated scheduling by scheduling the complete redundant executors, the adversary has memory (that is, the adversary can conduct memorization-based attacks) and then can continuously increase the attack success probability. That is, in the information entropy model, the attacker’s attack success probability increases with repeated scheduling, and the overall information entropy of the system decreases. The trend graph of the system information entropy in two cases is shown in Figure 4.

Before we present the information entropy-based metrics, we first give the following two assumptions:

**Assumption** **3.** *The heterogeneous redundant system A adopts the classical majority-consistent strategy for output adjudication.*

**Assumption** **4.** *There exists a state indicator T for a redundant executor *$a_{i}$*, which represents the adjustable interval of that redundant executor from completely risk-free to completely failed under the condition of having memorization-based attacks. *$p_{xt}(a_{i})$ *denotes the failure probability of the system in the t-th state of* $a_{i}$*,*  t∈(1,T)*, and* $p_{xt}(a_{i})$ *is a monotonically increasing function of the state variable t.*

Then, we assessed the total information entropy of the heterogeneous redundant system *A* being attacked by any redundant executor. That is, the total information entropy of the system is the sum of the information entropy of each redundant executor being attacked successfully, which can be expressed according to Equation (7).

(7) $$\begin{array}{ll} H(A) & =H(a_{1},\ldots ,a_{m})\\ & =-(\sum_{t=1}^{T}p_{xt}(a_{i})\log p_{xt}(a_{i})+\sum_{t=1}^{T}p_{xt}(a_{j}|a_{i})\log p_{xt}(a_{j}|a_{i})+\ldots )\\ & =-\sum_{i=1}^{m}\sum_{j=1}^{i}\sum_{t=1}^{k}\left(p_{xt}(a_{i}\right)f(s_{ij})\log(p_{xt}(a_{i})f(s_{ij}))) \end{array}$$

$$\begin{array}{c} s.t.[(n+1)/2]\leq m\leq n\\ 0\leq f(s_{ij})\leq 1 \end{array}$$

In Equation (7), when any redundant executor $a_{i}$ fails due to an attack, *H*($a_{i}$) is reduced to 0, and the information entropy of the system decreases. However, the entropy of a single redundant executor $a_{i}$ is not monotonically decreasing. With these discussions, we now present the three metrics:

**(Metric 1) The number of scheduling states of the redundancy resources/** **executors**

We choose **the number of scheduling states** *T* as the first metric, which can be used as a basis for the information entropy and the trend of the information entropy change. In addition, it can be used as a basis for analyzing metric normalization in comparison with other scheduling algorithms. See Section 5.

**(Metric 2)** **The information entropy value**

Equation (8) defines the uncertainty function $F_{PA}(t)$, which is a monotonically decreasing function in the range of t∈[1,T] and is positively correlated with *H*(*A*). $F_{PA}(t)$ is the **information entropy** metric of the system. By removing $p_{xt}(a_{i})$ from Equation (7), we obtain the computing formula of $F_{PA}(t)$, as shown in Equation (8).

(8) $$\begin{array}{ll} F_{PA}(t) & =-(\sum_{t=1}^{T}\log p_{xt}(a_{i})+\sum_{t=1}^{T}\log p_{xt}(a_{j}|a_{i})+\ldots )\\ & =-\sum_{i=1}^{m}\sum_{j=1}^{i}(f(s_{ij})\cdot \sum_{t=1}^{T}\log(p_{xt}(a_{i})f(s_{ij})) \end{array}$$

$$\begin{array}{c} s.t.[(n+1)/2]\leq m\leq n\\ 0\leq f(s_{ij})\leq 1 \end{array}$$

**(Metric 3) The decay rate of the information entropy value**

In Equation (8), $F_{PA}(t)$ can denote the information entropy value of the CMD system *A* at the moment of state *t*. The smaller $F_{PA}(t)$, the less reliable the system. Additionally, when $F_{PA}(t)$ is 0, the system becomes completely unreliable. In addition, we propose the **decay rate of the information entropy value** $F_{PA}(\Delta t)$ as the third evaluation metric for assessing the system’s reliability. $F_{PA}(\Delta t)$ can be expressed as in Equation (9).

(9) $$F_{PA}(\Delta t)=-\sum_{i=1}^{m}\sum_{j=1}^{i}(f(s_{ij})\cdot \log(\frac{p_{xt}+\Delta p_{x\Delta t}}{p_{xt}}))$$

A larger $F_{PA}(\Delta t)$ value indicates a larger decay rate in the system entropy information entropy value, i.e., the fewer times the system can cope with memorization-based attacks, and the lower the system’s resistance to memorized attacks.

## 4. Scheduling Algorithm Based on the Information Entropy and Game Model

This section first presents a game model to capture the relationships between attackers and defenders, then the algorithm for scheduling redundant executors is given.

### 4.1. Game Model

Consider the game state of the mimic defense system for both attackers and defenders, each aiming for benefits. The adversary wishes to destroy the system by attacking the redundant executors to gain benefits, while the defender wishes to analyze the adversary’s attack strategy to make corresponding defense scheduling and reduce the benefits gained by the attacker. The benefits of both sides are negatively correlated. In the process of strategy adjustment, when both the adversary and the defender cannot gain more benefits by adjusting the strategy, the game reaches equilibrium, at which time the adversary will give up the attack due to the inability to obtain the desired attack benefits.

In this model, whether the adversary launches a subsequent attack in any attack state *t* mainly depends on whether the probability of the adversary obtaining a gain satisfies its expectation when the state moves from *t* to *t* + 1. Therefore, we first present the game model based on the complete information, i.e., the benefit matrix under the full information. Then, by pointing out its weakness, we give the game model based on incomplete information, and based on this, the equilibrium point is solved.

#### 4.1.1. Game Model Based on Complete Information Conditions

Consider a game model G={Ω,T,P,U}.

(1) $\Omega =\{\Omega_{A},\Omega_{D}\}$ denotes the game participants: attacker $\Omega_{A}$ and defender $\Omega_{D}$.

(2) ${T=\{T}_{A}{,T}_{D}\}$ denotes the game strategy space, the attacker strategy $T_{A}=\{T_{A1},T_{A2}\}$, and the defender strategy $T_{D}=\{T_{D1},T_{D2}\}$. $T_{A1}$ means that the attacker performs an attack strategy, $T_{A2}$ means that the attacker performs a no-attack strategy, $T_{D1}$ means that the defender performs an active scheduling defense, and $T_{D2}$ means that the defender shuts down the system.

(3) $P=\{\alpha_{A},\beta_{D}\}$ denotes the game strategy execution probability space. The probability that the adversary attacks to execute the strategy is denoted as $\alpha_{A}$ and the probability that the defender executes the strategy is denoted as $\beta_{D}$. Thus, $\alpha_{A}=\{\alpha ,1-\alpha \}$ and $\beta_{D}=\{\beta ,1-\beta \}$.

(4) ${U=\{U}_{A}{,U}_{D}\}$ denotes the payoff space of the game participants. $U_{A}$ is the attacker’s payoff and $U_{D}$ is the defender’s payoff.

At the same time, we make the following assumptions:

**Assumption** **5.** *Both players of the game will execute the strategy only if they are sure that the payoff of the strategy is positive.*

The gain parameter symbols of the adversary and the defender in system *G* are shown in Table 1. For the heterogeneous redundant system *A* with *m* redundancy, consider the offensive and defensive game strategies when the number of failed redundancies *k* satisfies $k\;<\;[\frac{m+1}{2}]$ and $k\;\geq \;[\frac{m+1}{2}]$, respectively, and construct the payoff matrix under the full information condition, as shown in Table 3.

From the results of the payoff matrix in Table 3, it is evident that under the conditions of complete information, the adversary can achieve the maximum gain regardless of the strategy adopted by the defender. There is no equilibrium point. This further confirms that the DHR architecture, under the conditions of infinite resources, cannot ensure the stable operation of the system when facing a memorization-based attack on a definite target under limited resources.

#### 4.1.2. Game Model Based on Incomplete Information Conditions

##### Model Under the Condition of the Incomplete Information Game

Similarly in the game model G={Ω,T,P,U}. To obtain an equilibrium solution for the system, the information about *k* needs to be hidden, transforming the model into an incomplete information game. At the same time, although the system itself has two sets of equilibrium points—(*T_A_*_1_, *T_D_*_1_), (*T_A_*_2_, *T_D_*_2_) or (*T_A_*_2_, *T_D_*_1_), (*T_A_*_1_, *T_D_*_2_)—the defender must choose one. In other words, for the defender’s basic goal of maintaining the normal operation of the system, the equilibrium point needs to be among (*T_A_*_2_, *T_D_*_1_), (*T_A_*_1_, *T_D_*_2_) as much as possible.

By averaging the return expectations under the incomplete information condition, we can obtain the payoff matrix for this condition. This matrix is shown in Table 4.

##### Model Equilibrium Solving Under Incomplete Information Game

The equilibrium equation under the mixed strategy condition can be obtained using the return matrix in Table 3:

(10) ((1−λ)B−kb)α=(e−kb)(1−α)

(11) (λc−kd)β=(c−kd)(1−β)

From Equations (7) and (8), two equilibrium solutions can be obtained.

(12) $$\alpha =\left\{\frac{e-kb}{(1-\lambda )B-2kb+e},\frac{(1-\lambda )B-kb}{(1-\lambda )B-2kb+e}\right\}$$

(13) $$\beta =\left\{\frac{kd-c}{(1-\lambda )c+2kd},\frac{(2-\lambda )c+kd}{(1-\lambda )c+2kd}\right\}$$

It is necessary to put the desired equilibrium point at (*T_A_*_2_, *T_D_*_1_), so it is necessary to have the equilibrium solutions {α,β}.

(14) $$\frac{e-kb}{(1-\lambda )B-2kb+e}\geq \frac{(1-\lambda )B-kb}{(1-\lambda )B-2kb+e}$$

(15) $$\frac{kd-c}{(1-\lambda )c+2kd}\leq \frac{(2-\lambda )c+kd}{(1-\lambda )c+2kd}$$

That is, from Equations (14) and (15), the constraints on the desired equilibrium point can be expressed by Equation (16).

(16) e>(1−λ)B

For the adversary, when there exists a revenue constraint e>(1−λ)B, the adversary may not make an attack because of an insufficient revenue, and its strategic equilibrium point is α. But for the defender, as long as the adversary satisfies the constraint e>(1−λ)B and the defender performs defensive scheduling, there exists a strategic equilibrium point β.

##### Redundant Scheduling Constraint Solving Based on Incomplete Information Game Models

We now derive the relationship between attack basic gain e and information entropy by combining with the metric formulas in Section 3. Consider a set of *m* executors $\left\{a_{1}\ldots a_{m}\right\}$ with *T* states for each redundant executor. For any redundant executor $a_{i}$, it is randomly in the *t*-th state. We make the following two assumptions with the set:

**Assumption** **6.** *For the set of executors $\left\{a_{1}\ldots a_{m}\right\}$* *, the total benefit of a successful attack by the adversary is B. The metric of the information entropy value of $\left\{a_{1}\ldots a_{m}\right\}$* *under the condition of state t is $F_{PA}(t)$* *, and $B\approx F_{PA}(t)$.*

**Assumption** **7.** *The base gain when the adversary is not traced is a constant value e and e is much larger than the attack cost of a single redundant executor b. The base gain is average and equal for all the T states, denoted as $e(t_{0})$.*

Then, according to Equations (12) and (14), the equilibrium solution and constraints of the set of executors $\left\{a_{1}\ldots a_{m}\right\}$ for the attacker can be expressed by Equation (17).

(17) $$\begin{array}{c} \alpha =\frac{e-kb}{(1-\lambda )F_{PA}(t)-2kb+e}\\ s.t.e>(1-\lambda )F_{PA}(t) \end{array}$$

In general, the gains available to the attacking party increase gradually as the attack continues. At state *t*, $F_{PA}(t)=mb$ can be taken. Since the objective function of the equilibrium solution *α* is a decreasing function of $F_{PA}(t)$ in the range of *T*, the maximum value of the model under the satisfied condition can be obtained and the objective function maxα satisfies Equation (18).

(18) $$\max\alpha =\frac{e-kb}{(1-\lambda )mb-2kb+e}$$

Meanwhile, according to Assumption 7, combined with the constraints of Equation (17), we obtain the constraints shown in Equation (19).

(19) $$e(t_{0})=e(\Delta t)\geq (1-\lambda )F_{PA}(\Delta t)$$

From Equations (18) and (19), we calculate the limiting value $\underset{e/b\to m}{\lim}\max\alpha$ when the ratio of the base gain *e* to the single redundant executor gain *b* converges to the total number of executors *m*. The solution of $\underset{e/b\to m}{\lim}\max\alpha$ with respect to the number of attacked redundant executors *k* within its definition domain is [(m+1)/2] or [(m−1)/2]. That is, a scheduling algorithm is designed to make the attacker’s gain in any t-state close to [(m+1)/2] or [(m−1)/2], and to satisfy that the change in the base gain from the t-state to *t* + 1. The maximum attacker–defender equilibrium gain is guaranteed when the amount of state change satisfies Equation (19).

### 4.2. The Description of the Scheduling Algorithm

According to the analysis results in Section 4.1, we propose a redundant executor scheduling algorithm based on the information entropy of randomized weight: REWS (Random Entropy Weight Scheduling Algorithm).

**Figure 5:**
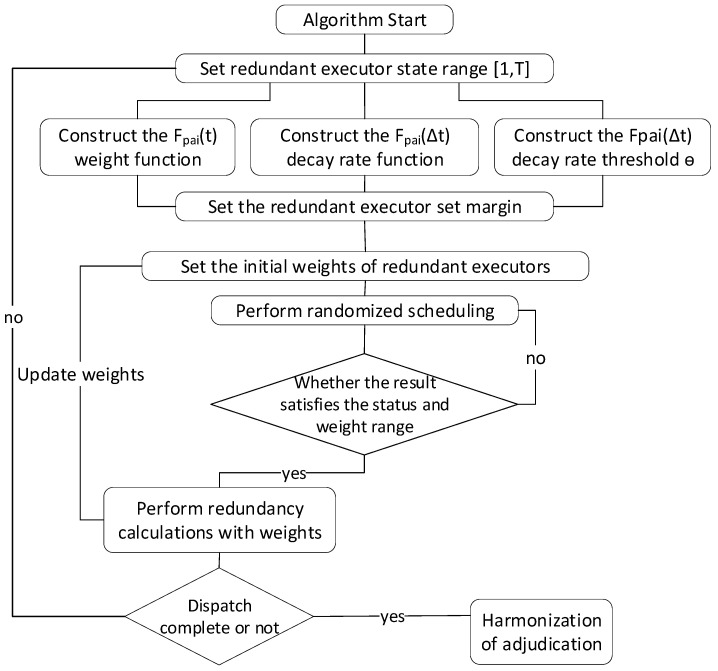
The flow chart of the algorithm.

The key steps of the algorithm are as follows. **Firstly**, select a collection of redundant executors, and construct a redundant executor’s attacked state value interval for each of them, which can be a separate indicator such as the number of attacks or the number of scheduling, or a composite indicator of multiple elements. Then, construct the state and information entropy weight function, as well as design the information entropy weight decay rate function when the state changes. **Next**, perform random scheduling of the set of redundant executors with weight random update feedback so that the updated sum of the weights of each redundant executor is in the vicinity of [(m+1)/2] or [(m−1)/2] information entropy weights. That is, the information entropy weights satisfy the maximum value of the attacker’s gain when the gains of the adversary and the defender are balanced. **Finally**, the calculation of the updated information entropy value makes the adversary unable to discern whether the attack result satisfies the gain or not, thus improving the reliability of the system. The flow chart of the algorithm is shown in Figure 5 and the three steps are detailed in the following:

**Step 1**. Initialization (**Algorithm 1**). First, in a redundancy pool with a margin of *n*, starting with the first redundant executor, set the range of the number of redundant executor states $\left\{t(a_{i})|t(a_{i})\in [1,T]\right\}$. Then, construct the weight function $w(F_{Pai}(t))$ of the redundant executor state and the information entropy value, construct the decay rate function $w(F_{Pai}(\Delta t))$ of the information entropy value when the state changes, and set the decay rate threshold of the information entropy value $e_{ai}(t_{0})$.

**Step 2**. Randomized scheduling with weight updates (**Algorithm 2**). First, determine the total redundancy *m* of the set of redundant executors for this scheduling, and perform a random scheduling computation in the pool of redundant bodies if the state intervals are satisfied and the decay rate of the information entropy value satisfies the threshold condition of $e_{ai}(t_{0})$. At each redundant executor scheduling, the adjudication result *V* is the product of each redundant executor’s computation result di and its weights cumulatively. The computational results *d_i_* are all compared with their previous settlement results, taking 1 if they are the same, and −1 if they are not.

Then, within the information entropy decay rate threshold, each redundant executor weight $w(F_{Pai}(t+1))$ that is scheduled is updated so that it is randomly fetched within the range of the offset $\varepsilon_{ai}$, i.e., it satisfies $w(F_{Pai}(t+1))\in [w(F_{Pai}(t+1))-\varepsilon_{ai},w(F_{Pai}(t+1))+\varepsilon_{ai}]$. The weights after taking values satisfy the conditions in Equation (20) or (21).

(20) $$\sum_{i=1}^{m}\max w(F_{Pai}(t+1))=\sum_{i=1}^{m}\left(w(F_{Pai}(t+1)+\varepsilon_{ai}\right)=w(\sum_{i=1}^{\left[(m+1)/2\right]}F_{Pai}(t))$$

(21) $$\sum_{i=1}^{m}\min w(F_{Pai}(t+1)=\sum_{i=1}^{m}\left(w(F_{Pai}(t+1)-\varepsilon_{ai}\right)=w(\sum_{i=1}^{\left[(m-1)/2\right]}F_{Pai}(t))$$

**Step 3**. If *a_i_* is not the last redundant executor, step 2 is repeated. Otherwise, a fictitious adjudication is performed based on the entropy weight value with the redundant executor calculation result. The resulting optimal *V* of the weight-based adjudication can indicate the reliability of the system as shown in Equation (22), *i* expresses the *i*-th redundant executor.

(22) $$\begin{array}{l} \max V=\sum_{i=1}^{m}w(F_{Pai}(t))d_{i}\\ s.t.\left\{\begin{array}{l} d_{i}=1,d_{i+1}=\left\{\begin{array}{c} 1,if\;d_{i+1}⊕d_{i}=1\\ -1,if\;d_{i+1}⊕d_{i}=0 \end{array}\right.\\ \sum_{i=1}^{m}\max w(F_{Pai}(t+1))=\sum_{i=1}^{m}\left(w(F_{Pai}(t+1)+\varepsilon_{ai}\right)=w(\sum_{i=1}^{\left[(m+1)/2\right]}F_{Pai}(t))\\ \sum_{i=1}^{m}\min w(F_{Pai}(t+1)=\sum_{i=1}^{m}\left(w(F_{Pai}(t+1)-\varepsilon_{ai}\right)=w(\sum_{i=1}^{\left[(m-1)/2\right]}F_{Pai}(t))\\ F_{Pai}(\Delta t)\leq e(t_{0}) \end{array}\right. \end{array}$$

**Algorithm 1** 
*Initialization*
 INPUT: redundancy pool *n*, Redundancy *n*, range of redundant executor states $\left\{t(a_{i})|t(a_{i})\in [1,T]\right\}$, information entropy weight function $w(F_{Pai}(t))$, decay rate function of the information entropy value $w(F_{Pai}(\Delta t))$, decay rate threshold of the information entropy value $e_{ai}(t_{0})$. OUTPUT: The set of redundant executor states *t*_*set*, initial weight set *w*_*set*, information entropy value decay rate set *rt*_*set*, and information entropy value decay rate threshold set *ct*_*set*. for 1 ≤ *I* ≤ *n* do:*t*_*set* = *t*_*set* + {$t(a_{i})$}*w*_*set* = *w*_*set* + {$w(F_{Pai}(t))$}*rt*_*set* = *rt*_*set* + {$F_{Pai}(\Delta t)$}*ct*_*set* = *ct*_*set* + {$e_{ai}(t_{0})$}endforoutput *t*_*set*,*w*_*set*,*rt*_*set*,*ct*_*set*


**Algorithm 2** *Randomized scheduling with weight updates* INPUT: Scheduled set of redundancies *A* = {$a_{1}\ldots a_{m}$}, Residual degree m of set *A*, Stochastic scheduling function *C*(*m*,*n*), the result of the redundant executor ai randomized scheduling *d*(*i*), the result set *D*, the redundant executor ai weight update offset value $\varepsilon_{ai}$.
 OUTPUT: mimetic adjudication result *V*. 
Initialization*D* = *C*(*m*,*n*)for *d*(*i*) in *D* do:if (*t*_*set*(*i*) > *T*) or (*rt*_*set*(*i*) > *ct*_*set*(*i*)) then*V* = nulloutput *V*breakend ifend forif *V* ≠ 0 thenfor *d*(*i*) in *D* do:if *d*(*i*) ⊕ *d*(0) = 1 then*V* = *V* + *w*_*set* (*i*)*d(*i*)else if *d*(*i*) ⊕*d*(0) = 0 then*V* = *V* + (−*w*_*set* (*i*))*d(*i*)end ifif *t*_*set* (*i*) + 1 ≤ *T* then*t*_*set* (*i*) = *t*_*set* (*i*) + 1if *w*_*set* (I + 1) > 0 then*w*_*set* (I + 1) = random ($w(F_{Pai}(t\text{\_}set(i+1)))\pm \varepsilon$)end ifend ifend forif (($\sum_{1}^{\left[(m+1)/2\right]}(w\text{\_}set(i))==\sum_{1}^{m}(w\text{\_}set(i+1))$) or $\sum_{1}^{\left[(m-1)/2\right]}(w\text{\_}set(i))==\sum_{1}^{m}(w\text{\_}set(i+1))$) and (*V* = null) thenoutput *V*elseoutput nullend ifend if


## 5. Simulation Evaluation

In this section, we perform simulations to evaluate the scheduling algorithm REWS under memorization-enabled attacker and finite resource qualification conditions, as well as the metrics (defined in Section 3) related to the information entropy value. The experiments are conducted using the system with an Intel Core i7 7200 CPU, 16 GB DDR memory, and a Windows 11 Professional operating system. The software running environment is Python 3.9.

### 5.1. Experiment Setup

The experiment initialization design mainly includes basic conditions initialization, index initialization, and algorithm model initialization, detailed in the following:

(i) Basic conditions initialization

It is mainly set for the redundant resource pool and the set of redundant executables. In this paper, we set a redundant pool with a redundancy *n* of 9. The similarity between the redundant executors is randomly generated with a β-distribution with parameters (5, 15), and then the similarity matrix $S_{ij}$ is obtained as shown in Equation (23).

(23) $$\left(\begin{array}{ccccccccc} 1 & 0.229 & 0.134 & 0.316 & 0.242 & 0.345 & 0.280 & 0.225 & 0.383\\ 0.229 & 1 & 0.125 & 0.215 & 0.209 & 0.302 & 0.210 & 0.069 & 0.153\\ 0.134 & 0.125 & 1 & 0.259 & 0.282 & 0.321 & 0.206 & 0.240 & 0.185\\ 0.316 & 0.215 & 0.259 & 1 & 0.404 & 0.139 & 0.358 & 0.165 & 0.181\\ 0.242 & 0.209 & 0.282 & 0.404 & 1 & 0.241 & 0.361 & 0.238 & 0.190\\ 0.345 & 0.302 & 0.321 & 0.139 & 0.241 & 1 & 0.280 & 0.270 & 0.319\\ 0.280 & 0.210 & 0.206 & 0.358 & 0.361 & 0.280 & 1 & 0.386 & 0.292\\ 0.225 & 0.069 & 0.240 & 0.165 & 0.238 & 0.270 & 0.386 & 1 & 0.264\\ 0.383 & 0.153 & 0.185 & 0.181 & 0.190 & 0.319 & 0.292 & 0.264 & 1 \end{array}\right)$$

At the same time, according to the relationship between the redundancy and the safety gain in the existing research, we select the redundant executor set margin m as 3 and 4 for the computational research.

(ii) Metric normalization for the assessment of dynamism

In order to compare the algorithms and metrics in the experiments with those in the established research, it is necessary to normalize the metrics proposed in this paper with the traditional metrics. Consider a mimic defense system *A*. Among the traditional research metrics, the scheduling cycle metric *T* is generally used to evaluate the dynamics of the system.

We investigate the mainstream scheduling algorithms proposed in recent years, including the heterogeneity-based CRS algorithm [13], the heterogeneity-based extension of the history confidence-based HDCD [31], and the HHAC algorithm [29]. The average number of scheduling times for a single redundant executor of the system during one scheduling cycle is about 3.6 and 8.9 times at residuals *m* = 3 and 4, respectively. Therefore, for the purpose of metric normalization, we adopt the average number of times that a single redundant executor is dispatched within one scheduling cycle of these three algorithms as the upper limit of the state range of the redundant executor.

(iii) Initialization of the information entropy weight-based scheduling model

According to the study in Section 3, $w_{t}=w(F_{Pai}(t))$ is a composite function of the attacked state of the redundant executor, where $F_{Pai}(t)$ is a monotonically decreasing function on *t*. Meanwhile, the function for when there is a memory attack and as *t* increases, the information that can be mined is decreasing gradually, i.e., $\frac{dH(A)}{dt}$ decreases gradually and $\underset{t\to \infty }{\lim}\frac{dH(A)}{dt}=0$. Therefore, in this paper, the function $w=e^{-\eta t}$ with similar properties is selected to replace the composite function for the approximate solution analysis. Where, η is the regulation parameter, *w* is the entropy weight, and *t* is the state of the redundant executor being attacked.

**Figure 6:**
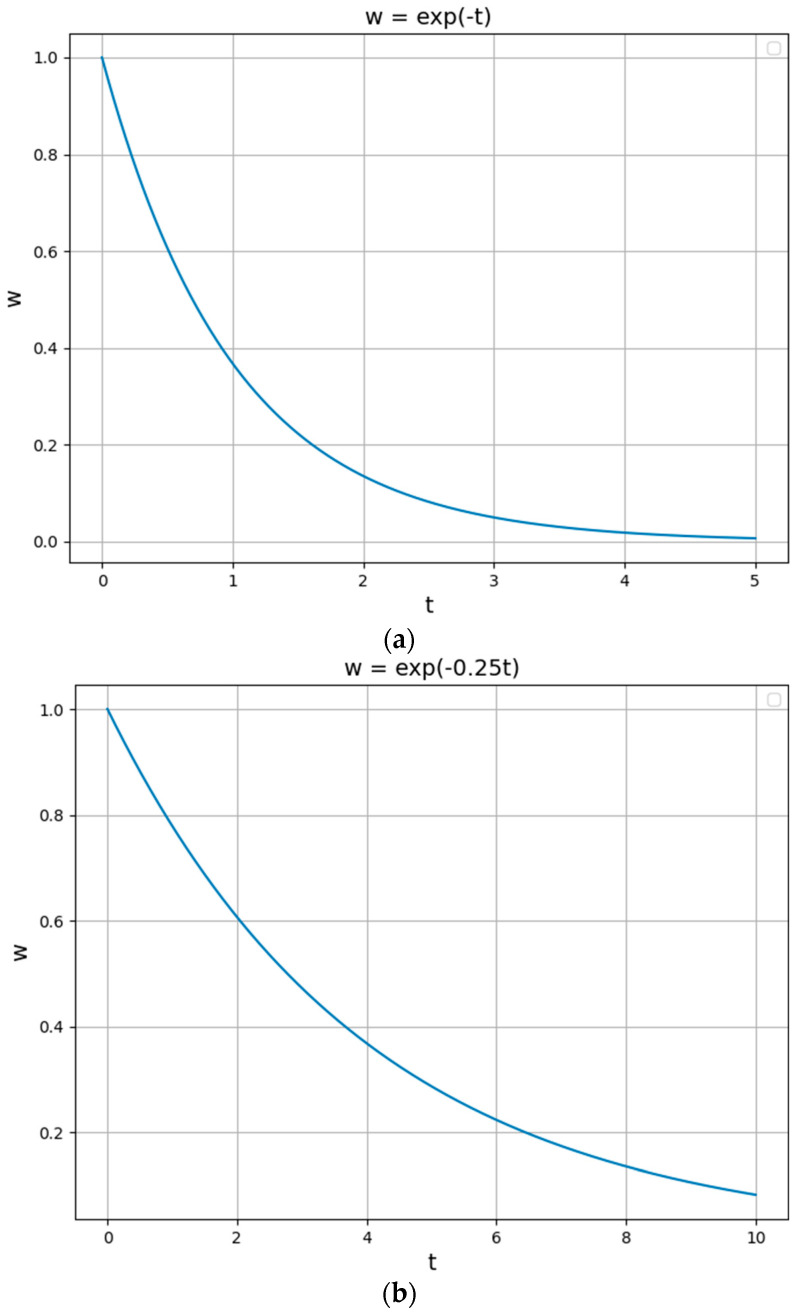
(**a**) Weights and scheduling time functions for η of 1. (**b**) Weights and scheduling times functions for η of 0.25.

Meanwhile, from the conclusion of the above study, it is necessary to control the range of the scheduling state values within 4.6 and 9.9 times when the margin *m* is taken as 3 and 4. Therefore, we take 1 and 0.25, respectively, and the results are shown in Figure 6.

The maximum number of states is approximated as 5 and 10 in both cases and the values of each scheduling weight are shown in Table 5 and Table 6.

Therefore, the weighting function parameters η can be taken as 0.5 and 0.25, respectively, and the decay rate thresholds θ for the overall information entropy value are 20% and 10%, respectively, for the comparison experiments.

### 5.2. Experimentation and Analysis of Algorithm Dynamics Under Limited Resource Conditions

We conduct simulation experiments to compare the CRS, HDCD, HHAC, and REWS algorithms under limited resources. To facilitate the comparison of the experimental results, this paper makes the following assumptions:

**Assumption** **8.** *All the redundant executor program sets cannot be repeated with the initial program.*

**Assumption** **9.** *Any redundancy set scheme that has been invoked is also unrepeatable (including HDCD, HHAC 2 algorithms with no increase in historical confidence or local confidence, Local Confidence (LC) and a decay rate of 100%).*

Under this condition, we conduct two experiments on dynamics, through which we expect to find the algorithm with the highest average scheduling period, the average number of states of the redundant executors, and the scheduling period to state ratio. Among them, the higher the average scheduling period, the stronger the system dynamics and the higher the reliability; a higher average number of states of the redundant executors indicates a higher initial information entropy value under the condition of limited resources, i.e., the stronger the reliability of the initial state; and the higher the ratio of the scheduling period to the state indicates that each scheduling under the condition of limited resources plays a bigger role in the reliability of the system. We conducted 100 independent experiments under simulated finite resource conditions and obtained the following results.

**Figure 7:**
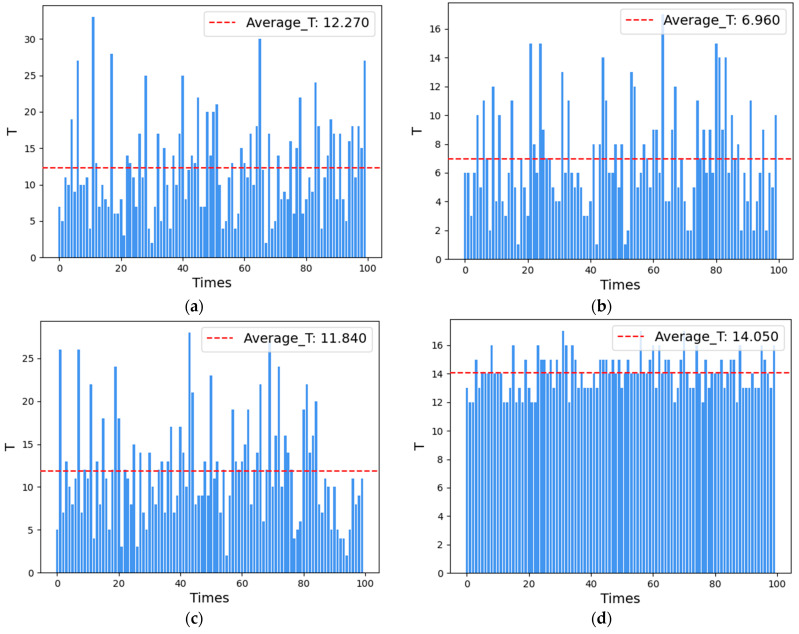
(**a**) CRS algorithm scheduling period for m = 3. (**b**) HCDC algorithm scheduling period for *m* = 3. (**c**) HHAC algorithm scheduling period for *m* = 3. (**d**) REWS algorithm scheduling period for *m* = 3.

(i) At *m* = 3, the REWS algorithm is chosen as the weighting function for the experiments. The scheduling period *T* of the CRS algorithm, the HDCD algorithm, the HHAC algorithm, and the REWS algorithm is shown in Figure 7a–d.

**Figure 8:**
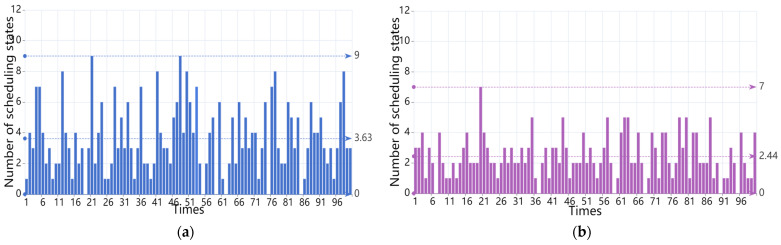

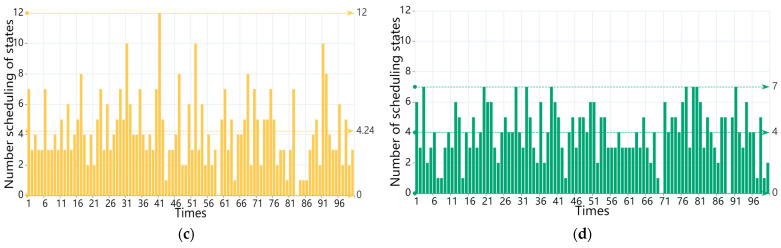
(**a**) Average number of scheduling states for the CRS algorithm for *m* = 3. (**b**) Average number of scheduling states for the HCDC algorithm for *m* = 3. (**c**) Average number of scheduling states for the HHAC algorithm for *m* = 3. (**d**) Average number of scheduling states for the REWS algorithm for m = 3.

**Figure 9:**
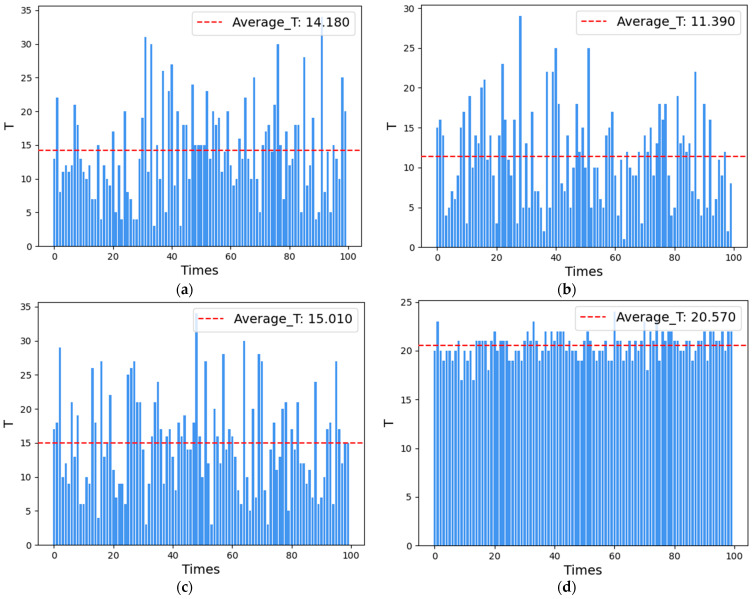
(**a**) CRS algorithm scheduling period for *m* = 4. (**b**) HCDC algorithm scheduling period for *m* = 4. (**c**) HHAC algorithm scheduling period for *m* = 4. (**d**) REWS algorithm scheduling period for *m* = 4.

The average number of scheduling states for the CRS algorithm, the HDCD algorithm, the HHAC algorithm, and the REWS algorithm for *m* = 3 is shown in Figure 8a–d.

(ii) The REWS algorithm selects $w=e^{-0.25t}$ as the weight function for the experiment at *m* = 4. The scheduling period T of the CRS algorithm, the HDCD algorithm, the HHAC algorithm, and the REWS algorithm is shown in Figure 9.

**Figure 10:**
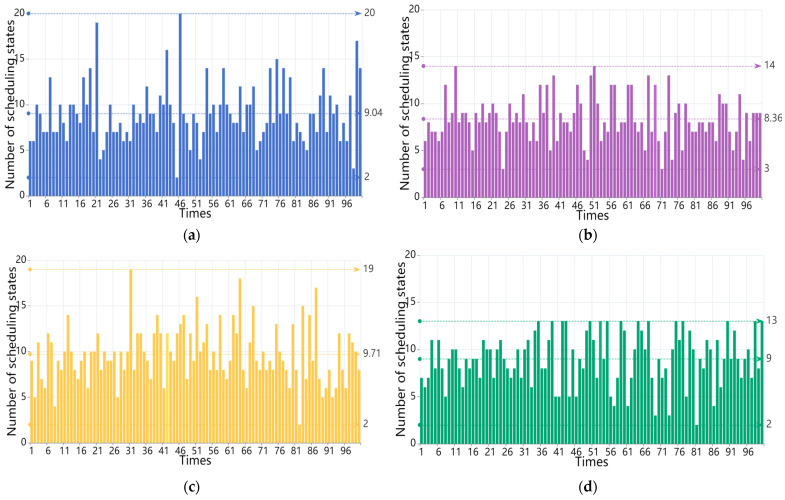
(**a**) Average number of scheduling states for the CRS algorithm for *m* = 4. (**b**) Average number of scheduling states for the HCDC algorithm for *m* = 4. (**c**) Average number of scheduling states for the HHAC algorithm for *m* = 4. (**d**) Average number of scheduling states for the REWS algorithm for *m* = 4.

The average number of scheduling states for the CRS algorithm, the HDCD algorithm, the HHAC algorithm, and the REWS algorithm for *m* = 4 is shown in Figure 10a–d.

The comparison of the experimental results is shown in Table 7.

From the experiments, it can be observed that the average scheduling period of the REWS algorithm is about 14.05 and 20.57 under the conditions of redundancy 3 and 4, respectively, even though the scheduling of the redundant system failure is about 14.05 and 20.47 times at this point. This result is 1.14 and 1.44 times more than the CRS algorithm, 2.01 and 1.8 times more than the HCDC algorithm, and 1.18 and 1.32 times more than the HHAC algorithm, respectively, and it also shows that the REWS algorithm has a better dynamic than the other algorithms under the condition of limited resources. Secondly, the average number of states of the REWS algorithm is 4 and 9, respectively, which is only slightly lower than the HHAC algorithm. This indicates that its initial reliability is better and only slightly worse than the HHAC algorithm. Finally, the period-to-state ratios of the REWS algorithm are 3.51 and 2.28, respectively, which are 1.03 and 1.45 times higher than those of the CRS algorithm, 1.23 and 1.67 times higher than those of the HCDC algorithm, and 1.17 and 1.43 times higher than those of the HHAC algorithm. This suggests that, even in the case of a less-than-optimal initial reliability, the scheduling of each time of the REWS algorithm plays a more significant role in the system reliability than the other algorithms. The system reliability is better than the other algorithms and the scheduling effectiveness of the system is higher.

### 5.3. Reliability Analysis Under a Memorization-Based Attack

**Figure 11:**
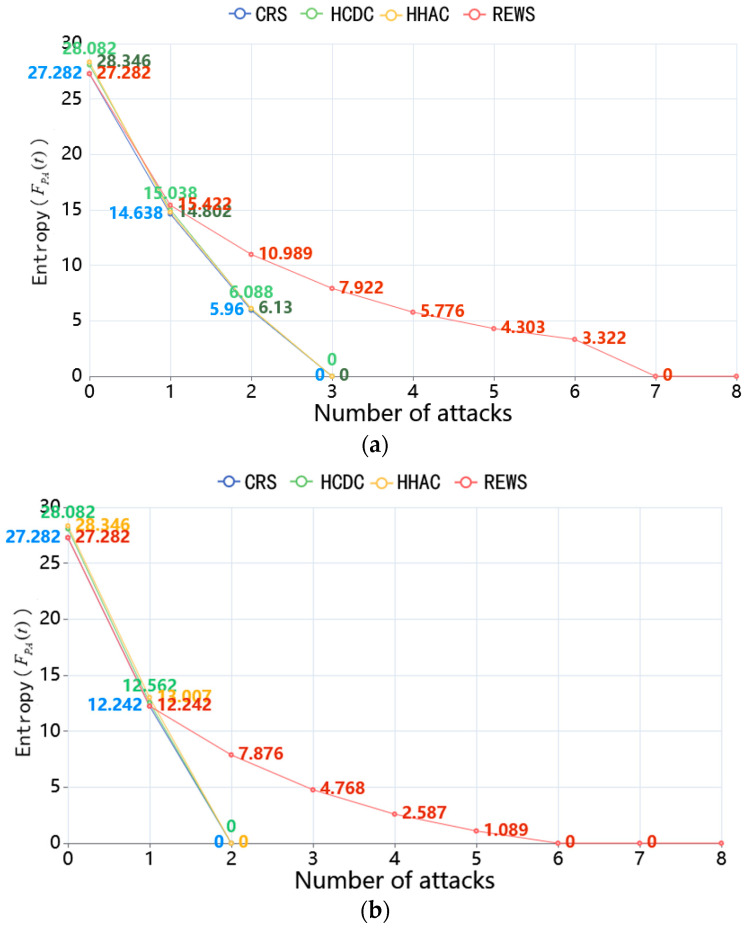
(**a**) Experiments on different algorithms against memorization-based attacks for *m* = 3. (**b**) Experiments on different algorithms against memorization-based attacks for *m* = 4.

For targeted memorization-based attacks, if an attacker discovers a high-threat 0-day vulnerability in a redundant executor, it is often difficult for the defender to quickly find an effective countermeasure. This means that all the scheduling strategies with respect to that redundant executor are at risk. Therefore, in this paper, we set the condition that a single redundant executor is unreliable after each scheduling, i.e., the decay rate of the information entropy is 100% to simulate the memorization-based attack for the experiments. We would like to experimentally find the algorithm that can resist the maximum number of memorized attacks under the condition of memorized attacks, and the experimental steps are specified as follows:

(i) First, still choosing the residual degree *m* = 3, 4, according to the similarity matrix constructed in Section 4.1, the average similarity of the above four algorithms is calculated as shown in Table 8.

(ii) In order to simplify the calculation, let $f(s_{ij})=s_{ij}$, then according to Equation (8), the initial information entropy values of the four algorithms can be calculated as shown in Table 9.

(iii) Finally, under the condition that the decay rate θ of the information entropy of a single redundant executor is 100% and according to the algorithmic process in Section 4, the scheduling is carried out at the residual degree of *m* = 3 and 4, respectively, then the trend of the information entropy is shown in Figure 11a,b.

From the experiments, it can be observed that the redundancy is permanently unreliable by the back side of the scheduling due to the memorization-based attack, which makes the traditional scheduling algorithms, such as the CRS algorithm, the HCDC algorithm, and the HHAC algorithm, unreliable by at most 3 and 2 attacks, respectively. As for the REWS algorithm, after the first scheduling, it can quickly adaptively adjust the adjudication strategy through the weights to make the attacker and the defender reach an equilibrium. This means that the subsequent scheduling in the case of the information entropy of the redundant executor that has been scheduled is 0, and it can still guarantee that the total information entropy value of the set of redundant executors is greater than 0, which in turn makes the whole system able to withstand 7 and 6 attacks to make the system unreliable.

### 5.4. Analysis of the Algorithm Time Complexity

According to the related literature, the time complexity of the CRS algorithm, the HCDC algorithm, the HHAC algorithm, and the main part of the REWS algorithm is shown in Table 10.

The CRS algorithm has the smallest time complexity of $o(n^{2})$, the REWS algorithm has a slightly larger time complexity than the CRS algorithm and a smaller time complexity than the HCDC algorithm and the HHAC algorithm, which is o(n(n×t)), and the HCDC algorithm and the HHAC algorithm have the largest time complexity of $o(n^{2}\times Y)$ and $o(n(n^{2}+(n-1)n))$, respectively. Here, *t* is the number of scheduling times determined according to the different weight functions and *Y* is the number of redundant executor scheduling times based on the historical confidence.

## 6. Conclusions and Future Work

This paper aims to address the problem of a gradual decrease in the reliability of the scheduling cycle in the face of limited scheduling resources and a memorization-based attack environment. Based on the mimic defense system of the DHR architecture in the cloud service platform of the railroad internal service network, relying on the actual engineering and construction scenarios of the CRCC, we propose three reliability evaluation metrics of the CRCC-DHR architecture in terms of the number of scheduling states of the redundancy executors, the information entropy value, and the decay rate of the information entropy value. On this basis, the problem is modeled and solved based on the incomplete information game model, and at the same time, a random entropy weight redundant executor scheduling algorithm, namely, the REWS algorithm, is further proposed.

Then, based on the evaluation metrics proposed in this paper, simulations are conducted to verify and compare the dynamics of the system and the reliability capability against memorization-based attacks of the REWS algorithm, the CRS algorithm, the HCDC algorithm, and the HHAC algorithm under the conditions of a residual degree of 3 and 4. The experimental analysis shows that the average scheduling period of the REWS algorithm is 1.14 and 1.44 times that of the CRS algorithm, 2.01 and 1.8 times that of the HCDC algorithm, and 1.18 and 1.32 times that of the HHAC algorithm, respectively, under the conditions of residuals 3 and 4, i.e., it means that the REWS algorithm is a better dynamic under the conditions of limited resources. Meanwhile, the cycle state ratio of the REWS algorithm is 1.03 and 1.45 times the CRS algorithm, 1.23 and 1.67 times the HCDC algorithm, and 1.17 and 1.43 times the HHAC algorithm, which means that each scheduling of the REWS algorithm plays a greater role in the system reliability and the scheduling effectiveness of the system is higher. In terms of the reliability against memorization-based attacks, under the condition of margins of 3 and 4, the REWS algorithm can withstand 7 and 6 attacks, respectively, to make the system unreliable, while the traditional scheduling algorithm can be made unreliable at most 3 and 2 attacks, respectively, i.e., its reliability is higher due to the traditional scheduling algorithm.

Although the REWS algorithm improves the reliability of the DHR architecture under the conditions of limited scheduling resources and memorization-based attacks, the total information entropy of the system still gradually decreases. Further improvements to the REWS algorithm will be explored in future research, focusing on enhancing the information entropy model. In addition, scalability is a big limitation of our scheduling algorithm. We will explore how to deal with this limitation and will also explore the reinforcement learning technique to design the scheduling algorithm. Moreover, we will explore how to collect data in the CRCC-DHR, establish a similarity matrix, and verify the effectiveness of our algorithm.

## Figures and Tables

**Table 1:** Comparison of different technical characteristics (“√” indicates that the condition is satisfied).

Approach	Primary Model	Features			Fault Tolerance
		Dynamic	Heterogeneity	Redundancy	
BFT	Master-slave			√	3*f* – 1
FTR	Master-slave			√	*f + 1*, *2f*, *3f…*
MTD	Master-slave	√		√	*f + 1*, *2f*, *3f…* or time redundancy
CMD	customizable	√	√	√	customizable

**Table 2:** Symbol definition.

Symbol	Definition
*A*	*A* mimic defense system
$a_{i}$	The *i*-th redundant resources/executors in *A*
*n*	The number of redundant resources/executors in redundant resource pools
*m*	The number of redundant executors in a scheduling process
*k*	The number of failed redundancies in system *A*
*p*($a_{i}$)	Probability that the redundant executor $a_{i}$ is disabled by an attack
$p{\left(a_{i}\ldots a_{l}\right)}_{l-i}$	Jointly distributed probability of the failure of *l-i* redundancies together
*p*(*A*)*_m_*	The failure probability of system *A* with a scheduling redundancy of *m*
$p_{xt}$	The probability that $x_{t}$ pieces of information are available
$F(p_{xt})$	The uncertain function of the probability of the occurrence of $x_{t}$
*t*	The *t*-th scheduling state, 1 ≤ *t* ≤ *T*
*T*	The number of (scheduling) states
$F_{PA}(t)$	The information entropy metric of system *A*
$F_{PA}(\Delta t)$	The decay rate of the information entropy value
*b*	Attack cost for an adversary *b* > 0
*c*	Defender’s base gain when the system is functioning normally, *c* > 0
*e*	Attacker’s base gain when undetected, *e* > 0
*d*	Cost to the defender when scheduling 1 redundant executor, 0 < *nd* ≤ *c*
*B*	Total gain from a successful attack by an attacker, 0 < *nb* ≤ *B*
λ	Proportionality parameter of return expectations for different ranges of values of *k*, 0 < *λ* < 1
{α,β}	Equilibrium solution of the game model
*t*($a_{i}$)	Attacked state of the redundant executor *a_i_*
$w(F_{Pai}(t))$	Information entropy weight function of the redundant executor $a_{i}$ in state *t*
$w(F_{Pai}(\Delta t))$	The rate of change of the information entropy weights when the redundant executor $a_{i}$ changes from state *t_i_* to state *t_i_* + 1
$e_{ai}(t_{0})$	Decay rate threshold for information entropy values
*d_i_*	Classification of computational results for redundant executor $a_{i}$
$\varepsilon_{ai}$	Randomized offset values for redundant executor $a_{i}$ weights
*V*	Mimetic adjudication results

**Table 3:** Payoff matrix under full information.

*k* < [(*n* + 1)/2]			*k* ≥ [(*n* + 1)/2]	
	*T*_*D*1_	*T*_*D*2_	*T*_*D*1_	*T*_*D*2_
***T*_*A*1_**	(−*kb*,*c*−*kd*)	(*e*−*kb*,0)	(*B*−*kb*, −*kd*)	(*e*−*kb*,0)
***T*_*A*2_**	(*e*,*c*−*kd*)	(*e*,0)	(*e*,*c*−*kd*)	(*e*,0)

**Table 4:** Payoff matrix under incomplete information.

*k* Unknown		
	*T_D_*_1_	*T_D_*_2_
***T_A_*_1_**	((1−λ)B−kb,λc−kd)	(*e − kb*,0)
***T_A_*_2_**	(*e,c − kd*)	(*e*,0)

**Table 5:** The entropy weights are taken at $w=e^{-t}$.

*T*	*w*
1	0.37
2	0.15
3	0.05
4	0.02
5	approximately equal to 0

**Table 6:** The entropy weights are taken at $w=e^{-0.25t}$.

*t*	*w*
1	0.78
2	0.61
3	0.47
4	0.37
5	0.29
6	0.22
7	0.17
8	0.14
9	0.11
10	approximately equal to 0

**Table 7:** Different scheduling algorithms scheduling **cycle** *T*. Average number of scheduling states, *n*; period-to-state ratio, *T*/*n*.

	Redundancy *m* = 3			Redundancy *m* = 4		
	Scheduling Cycle *T*	Average Number of Scheduling States *n*	Period-to-State Ratio *T*/*n*	Scheduling Cycle *T*	Average Number of Scheduling States *n*	Period-to-State Ratio *T*/*n*
CRS	12.27	3.63	3.38	14.26	9.04	1.57
HCDC	6.96	2.44	2.85	11.39	8.36	1.36
HHAC	11.84	4.24	3.01	15.55	9.71	1.60
REWS	**14.05**	**4.00**	**3.51**	**20.57**	**9.00**	**2.28**

**Table 8:** Average similarity of the four different scheduling algorithms.

Average Similarity	CRS	HCDC	HHAC	REWS
*m* = 3	0.254	0.170	0.144	0.254
*m* = 4	0.244	0.191	0.171	0.240

**Table 9:** Information entropy value of the different scheduling algorithms.

	CRS	HCDC	HHAC	REWS
Information entropy value	27.282	28.082	28.346	27.282

**Table 10:** The time complexity of the four different scheduling algorithms.

	CRS	HCDC	HHAC	REWS
Time complexity	$o(n^{2})$	$o(n^{2}\times Y)$	$o(n(n^{2}+(n-1)n))$	o(n(n×t))

## Data Availability

No new data were created or analyzed in this study. Data sharing is not applicable to this article.
